# A 9-year analysis of medical malpractice litigations in coronary artery bypass grafting in China

**DOI:** 10.1186/s13019-023-02172-x

**Published:** 2023-02-12

**Authors:** Jie Chen, Tianyi Zhang, Dan Feng, Yuehui Liu, Tao Zhang, Jingtong Wang, Lihua Liu

**Affiliations:** 1 Medical School of Chinese People’s Liberation Army, Beijing, China; 2 Institution of Hospital Management, Medical Innovation Research Division of Chinese PLA General Hospital, Beijing, China; 3 Department of Medical Quality Management, Peking University People’s Hospital, Beijing, China; 4 Department of Vascular Surgery, Peking University People’s Hospital, Beijing, China

**Keywords:** Coronary artery bypass, Malpractice, Postoperative complications

## Abstract

**Background:**

The coronary artery bypass grafting (CABG) is one of the high-risk litigated medical specialties. Further elucidating the causes behind these malpractice claims can help physicians avoid patient injury. This study analyzed CABG litigations occurred in different level hospitals to outline the basic characteristics, as well as present a analysis on the medical malpractice that result in lawsuits.

**Methods:**

This study utilized the “China Judgments Online” database to compile litigations from 2012 to 2021 across China. 109 cases related to the CABG were included in the study, and were analyzed for demographic, patient outcomes and verdict characteristics in different levels of hospitals.

**Results:**

The median age of plaintiff patient was 62 years, the median length of stay was 25 days, and the median responsibility ratio of the litigation cases was 30%. The average proportion of responsibility of national, provincial and municipal hospitals were 29.6%, 28.4% and 39.5% respectively, and the median days after surgery to death of that were 15, 9 and 5 separately. The top 5 postoperative complications in dispute cases were: low cardiac output syndrome, postoperative hemorrhage, non-surgical site infections, surgical site infections and arrhythmia.

**Conclusions:**

The diagnosis and treatment capabilities of coronary artery bypass grafting in different levels of hospitals in China were inconsistent, and the treatment capabilities in prefecture-level hospitals were lower than that in national hospitals. The procedural error, failure to properly monitor the patient and diagnostic errors were common in CABG litigations. Postoperative complications related to surgical injuries and insufficient basic postoperative management lead to a higher responsibility proportion.

## Key Points

1. The procedural error was the most common reason in CABG malpractice claims.
2. Malpractice occurred most in postoperative management procedure.
3. CABG claims caused by surgical injuries have a higher proportion of liability.

## Introduction

Ischemic heart disease is the leading cause of death worldwide, and coronary artery bypass grafting (CABG) is one of most commonly performed procedures to treat this disease. There were more than 160,000 procedures in the United States and 30,000 cases in China in 2017, and that carries an in-hospital mortality rate of 1.8–2.3% in the United States and China. Furthermore, the incidence of postoperative surgical complications continues to be high [1, 2]. The unsatisfied outcomes of the procedure usually cause litigations, and cardiothoracic surgery of which coronary artery bypass grafting (CABG) constitutes a substantial portion is one of the high-risk litigated medical specialties [3, 4].

On the other hand, the quality of health care called for more comprehensively description and evaluation. The medical quality index monitoring can obtain the incidence of adverse outcome events and postoperative surgical complications, and their severity can also be reflected through the mortality, postoperative hospital stay, medical expenses and other indicators [5, 6]. However, it is difficult to deduce the role of human error in the occurrence of these adverse events through daily quality monitoring. Medical malpractice litigations are another platform for clinicians to understand the deep causes of each adverse event and the proportion of human-avoidable factors contributing to this adverse outcome [7, 8].

To further elucidate the causes behind these malpractice claims can help physicians avoid patient injury and decrease liability risk. This study analyzed CABG litigations to outline the basic characteristics of that occurred in different level hospitals, as well as present a detailed analysis on common injuries and medical quality issues that result in lawsuits.

## Methods

Medical malpractice claims are described as lawsuits against health-care provider for patients’ injury or death arising from medical care [9]. The present research conducted a retrospective study to review the medical malpractice verdicts to analyze the harm on the patients who suffered the coronary artery bypass grafting (CABG) during 2012–2021 in China.

### Data source and inclusion criteria

The medical dispute judgment documents were searched from the database “China Judgments Online”, which was maintained by the Supreme Court of China, when included the phrase “medical malpractice” and “coronary artery bypass grafting”. While the medical dispute judgment documents were excluded under the following circumstance: (1) A medical dispute case may go through multiple rounds of litigation, such as the first instance, the second instance, and the final instance. In this instance, this study only retains the last judgment document; (2) “Coronary artery bypass grafting” was not the cause of this lawsuit, such as: performing as a medical history or conducting as an emergency salvage surgery for other procedures; (3) The medical litigation cases without valid medicolegal expert opinions; (4) Dispute cases for no medical malpractice judged by the court. According to the above inclusion and exclusion criteria, 109 cases related to coronary artery bypass grafting were included in the study.

Institutional review board approval was not requested or obtained because we used database information with public accessibility. Therefore, no written informed consent was required.

### Data collection

Data were extracted from the medical malpractice verdicts, including the patients’ gender, age, admission time, discharge time, surgery time, death time (if someone dead), complications and outcome of the injury, the level of involved medical institution, location of the court, results of judgments, proportion of liability.

### Statistical analysis

Descriptive statistics were used to evaluate the data. Data were presented as mean ± standard deviation (SD) or median ± interquartile range and percentages (%), analyzed by Cochran mantel Haenszel classification variable test, analysis of variance and rank sum test according to the type of variables. The gender, patient outcomes, length of stay, responsibility proportion of medical malpractice in different levels of hospitals were compared. Statistical significance was considered *p* < 0.05. Statistics were performed using R version 4.0.1.

## Results

A total of 109 cases met our inclusion criteria. Most plaintiffs patient were male (83 of 109), and patient age ranged from 44 to 81 years old, the median age was 62 years old (including 1 missing values), and the median length of stay was 25 days. The median responsibility ratio of the cases was 30%, of which 80 patients died, 18 were disabled, 7 were hospitalized longer, and 3 simply increased the cost of treatment (Table 1).

**Table 1:** Demographic data and characteristics of CABG malpractice claims (Subdivided According to Hospitals with Different Level) (Created by the authors)

Variables	Level of hospital					
	National	Provincial	Municipal	Test name	Statistics	*p*
*Gender (N,%)*						
Male	18(66.7)	34(79.1)	31(79.5)	CMH test	1.67	0.433
Female	9(33.3)	9(20.9)	8(20.5)			
Total	27(100.0)	43(100.0)	39(100.0)			
*Age*						
N(Missing)	27(0)	42(1)	39(0)	Rank sum test	H = 1.27	0.531
Median	65.0	65.5	62.0			
Q1, Q3	60.0, 77.0	60.0, 72.5	59.0, 69.0			
Min,Max	55.0, 81.0	44.0, 79.0	33.0, 77.0			
*Outcomes (N,%)*						
Death	17(63.0)	33(76.7)	30(76.9)	CMH test	2.27	0.322
Disability	8(29.6)	6(14.0)	5(12.8)			
Prolonged hospitalization	0(0.0)	3(7.0)	4(10.3)			
Increased medical costs	2(7.4)	1(2.3)	0(0.0)			
Total	27(100.0)	43(100.0)	39(100.0)			
*Preoperative hospital stay (days)*						
N(Missing)	27(0)	43(0)	39(2)	Rank sum test	H = 1.70	0.426
Median	8.0	10.0	13.0			
Q1, Q3	5.0, 16.0	5.0, 16.0	7.0, 18.0			
Min, Max	1.0, 30.0	1.0, 45.0	3.0, 31.0			
*Hospitalization days (days)*						
N(Missing)	27(0)	43(0)	39(0)	Rank sum test	H = 0.62	0.735
Median	26.0	22.0	25.0			
Q1, Q3	16.5, 41.0	12.5, 39.0	14.0, 35.0			
Min, Max	5.0, 136.0	2.0, 358.0	3.0, 154.0			
*Responsibility proportion for medical malpractice (%)*						
N(Missing)	27(0)	43(0)	39(0)	Rank sum test	H = 8.37	0.015*
Median	30.0	25.0	40.0			
Q1, Q3	15.0, 40.0	15.0, 40.0	30.0, 50.0			
Min, Max	0, 100.0	0, 70.0	0, 100.0			

*represents that the *p* value < 0.05

### Characteristics of CABG claims in different level of hospitals

There are different level of hospitals in China. National-level hospitals are usually under the supervision of the National Health Commission directly, or hospitals affiliated with the National Medical Center, or those affiliated to China’s top medical universities. A provincial-level hospital refers to a regional medical center in a province of China. It is a top hospital within the province, which usually registered with the Provincial Center for medical research or medical quality control. Municipal-level hospitals are medical institutions that primarily serve the regional population of a certain city.

There were 27 cases of primary consultation in National-level hospitals, of which 17 were deaths, the median age of patients was 65 years old, the median length of stay was 26 days, and the average proportion of responsibility was 29.6% ± 20.8%. The provincial-level hospitals involved 43 cases, of which 33 cases of death, the median age of the patients was 65.5 years old, the median length of stay was 22 days, and the average responsibility ratio was 28.4% ± 18.0%. While the municipal-level hospitals involved 39 cases, of which 30 cases of death, the median age of patients was 62 years old, the median number of hospital days was 25 days, the average responsibility ratio was 39.5% ± 18.7%. There were statistical differences in the proportion of responsibility for disputes in different levels of medical institutions (*p* < 0.05), and the proportion of responsibility for related cases in prefecture and municipal medical institutions is higher than that of state-level and provincial medical institutions (Table 1).

### Characteristics of cases prosecuted for the patient death

As the majority of the sample cases were caused by the death of patients (73.4%), this study analyzed the characteristics of this kind of cases (Table 2). The indicators of length of stay of deceased patients were analyzed in depth, including the total hospitalization days, preoperative hospitalization days and postoperative death days. The postoperative death days refers to the number of days spent in hospital between the end of surgery and the death of a patient, an indicator that reflected the level and ability of medical institutions to a certain extent. There were statistical differences in the “postoperative death days” in different levels of hospitals (*p* < 0.05), the median days after surgery to death in prefecture-level hospitals was 5, while that in provincial hospitals was 9 and in national hospitals was 15 days. The median of total hospitalization days of claims with patient death in national hospitals was shorter than that in all types of claims; meanwhile the liability ratio was higher. Furthermore, as with all case data, there were statistical differences in the proportion of compensation in different levels of hospitals in claims with patient death.

**Table 2:** Characteristics of CABG malpractice claims with patient death in the different levels of hospitals (Created by the authors)

Variables	Level of hospital					
	National	Provincial	Municipal	Test name	Statistics	*p*
*Gender (N,%)*						
Male	18(66.67)	24(85.71)	22(78.57)	CMH test	0.890	0.641
Female	9(33.33)	4(14.29)	6(21.43)			
Total	17(100.00)	33(100.00)	30(100.00)			
*Age*						
N(Missing)	17(0)	33(0)	30(0)	Rank sum test	H = 0.37	0.832
Median	62.0	62.0	63.0			
Q1, Q3	60.0, 69.0	60.0, 68.0	60.0, 67.8			
Min, Max	53.0, 81.0	46.0, 79.0	33.0, 77.0			
*Preoperative hospitalization days*						
N(Missing)	17(0)	33(0)	30(1)	Rank sum test	H = 2.29	0.317
Median	8.0	11.0	14.0			
Q1, Q3	6.0, 13.0	7.0, 16.0	6.0, 19.0			
Min, Max	3.0, 30.0	1.0, 45.0	3.0, 31.0			
*Length of stay (days)*						
N(Missing)	17(0)	33(0)	30(0)	Rank sum test	H = 0.44	0.805
Median	21.0	20.0	21.5			
Q1, Q3	16.0, 36.0	10.0, 36.0	10.3, 32.8			
Min, Max	5.0, 113.0	2.0, 129.0	3.0, 51.0			
*Postoperative death days*						
N(Missing)	17(0)	33(0)	30(0)	Rank sum test	H = 6.12	0.047*
Median	15.0	9.0	5.0			
Q1, Q3	5.0, 27.0	2.0, 26.5	1.0, 16.5			
Min, Max	2.0, 429.0	0.0, 196.0	0.0, 731.0			
*Responsibility proportion for medical malpractice (%)*						
N(Missing)	17(0)	33(0)	30(0)	Rank sum test	H = 4.77	0.092
Median	30.0	30.0	40.0			
Q1, Q3	20.0, 40.0	20.0, 40.0	30.0, 50.0			
Min, Max	10.0, 60.0	0.0, 63.0	0.0, 70.0			

*represents that the *p* value < 0.05

### Medical issues in CABG malpractice claims

**Fig. 1:**
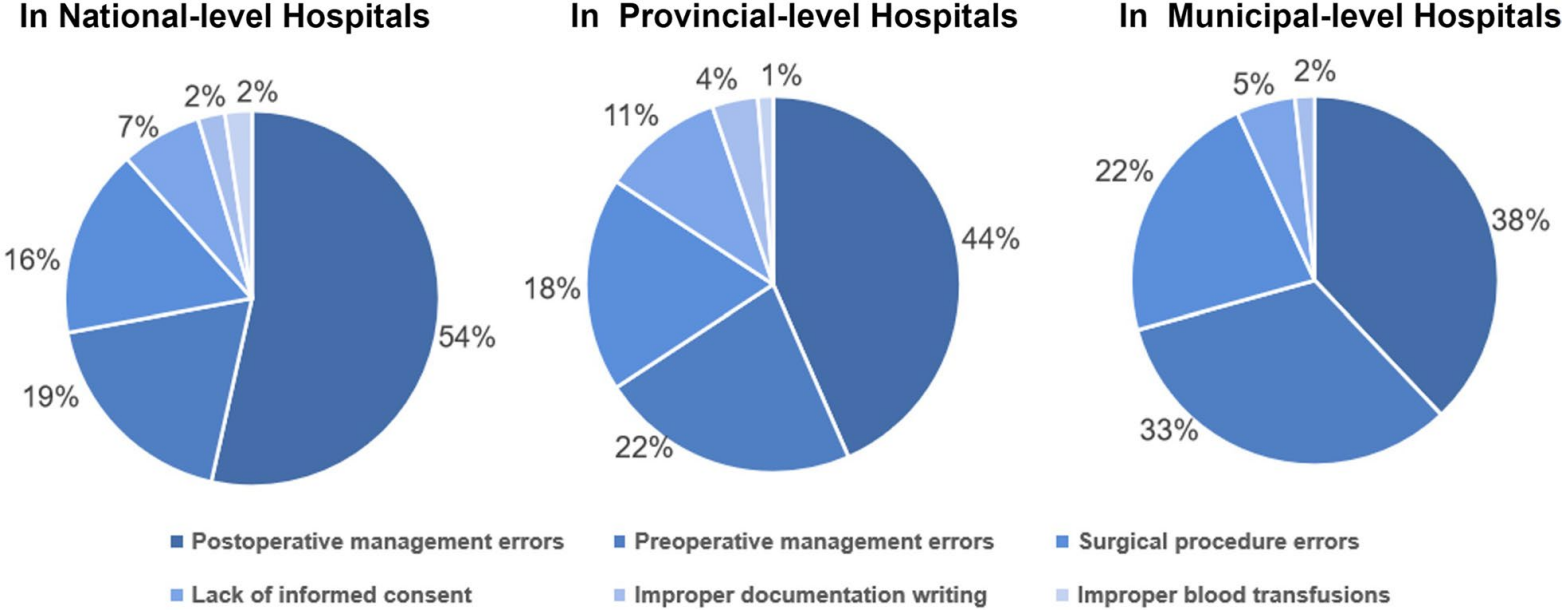
Distribution of medical issues in different types of hospitals. This figure reveals the distribution characters of each kind of Medical Issues in CABG medical claims occurred in different types of Hospital

The most common basis for litigation were postoperative management errors, which were involved in 71.6% claims, and the next most common one was preoperative management errors (40.4%), followed by the surgical procedure errors (31.2%), lack of informed consent (12.8%), improper documentation writing (4.6%) and blood transfusions (1.8%). Although this rule of occurrence was basically the same in hospitals at all levels, the relative proportions of each type of medical problem was not exactly the same at different levels of hospitals, with 54% of national hospitals having postoperative management problems, 19% having insufficient preoperative assessment accounts, and 16% having surgical procedures. In provincial-level hospitals, postoperative management problems accounted for 44%, insufficient preoperative assessment for 22%, and improper surgical procedures for 18%. Among municipal-level hospitals, 38% had postoperative management problems, 33% had insufficient preoperative evaluation, and 22% had improper surgical procedures (Fig. 1).

The common associated medical issues in these medical procedures were listed in Table 3. Many studies on the medical claims classified the misadventure into some abstract categories (e.g. improper performance of a procedure) [9–11], while, this study tied to describe the medical issues as concretely as possible to help reduce such errors in the future. For example, as to the postoperative procedure, the associated medical issues included failure to supervise or monitor a case, failure to recognize or misdiagnose complications, missed or delayed consultation, missed or false clinical examination, delayed treatment, performed when not indicated or contraindicated, medication or enteral nutrition errors, improper nursing care and premature discharged. During the preoperative procedure, failure to examine the following clinical tests could result in patient harm and claims, including the lower extremity vascular status, lung function, renal function, coagulation function, hypertension, and cardiac function examination. Because there were more than one error in a case usually, this study with a limited sample size didn’t not describe the proportion and the liability ratio of each problem.

**Table 3:** Medical issues in CABG malpractice claims by procedure group (Created by the authors)

Medical procedure	No.(%)	Associated medical malpractice
Postoperative management	78 (71.6%)	Failure to supervise or monitor a case Failure to recognize or misdiagnose complications No or delayed consultation Missed or false clinical examination Delay in treatment Performed when not indicated or contraindicated Medication or enteral nutrition errors Improper nursing care Premature discharged
Preoperative management	44 (40.4%)	Inadequate assessment Missed or false clinical examination: vascular status of lower extremities, pulmonary function, renal function, coagulopathy, hypertension, cardiac function misdiagnosed comorbidities: hypertrophic cardiomyopathy, arteriosclerosis obliterans of lower extremities, lung space occupying Improper operation scheme: Non indication surgery, improper timing of surgery, wrong surgical procedure, inappropriate selection of bridge blood vessels, too many items for one surgery, combined with other non-essential surgery, missing lesions Uncontrolled operative contraindications, Medication Errors: use of contraindicated medications, Inadequate duration of anticoagulation discontinuation Implant specification error
Surgical procedure	34 (31.2%)	Improper surgical procedures: bleeding, anastomotic leak, poor sternal fixation, visceral injury, bridging vessel stenosis Surgical foreign body left in patient after procedure Improper anesthesia monitoring: Intraoperative hypotension, difficulties with anesthesia resuscitation Medication Errors: Inadequate neutralization of anticoagulants
Informed consent	14 (12.8%)	Inadequate informed consent: Surgical complications, surgical procedure, autopsy matters, alternative treatment options, precautions for medication(anticoagulant), follow-up examinations
Documentation writing	5 (4.6%)	Tampering medical records, irregular modification of medical records, omission of medical records, in vivo implant information not entered medical records, not signed informed consent for treatment
Blood transfusions	2 (1.8%)	Bloodborne infectious diseases

### Analysis of postoperative complications in CABG medical litigations

**Fig. 2:**
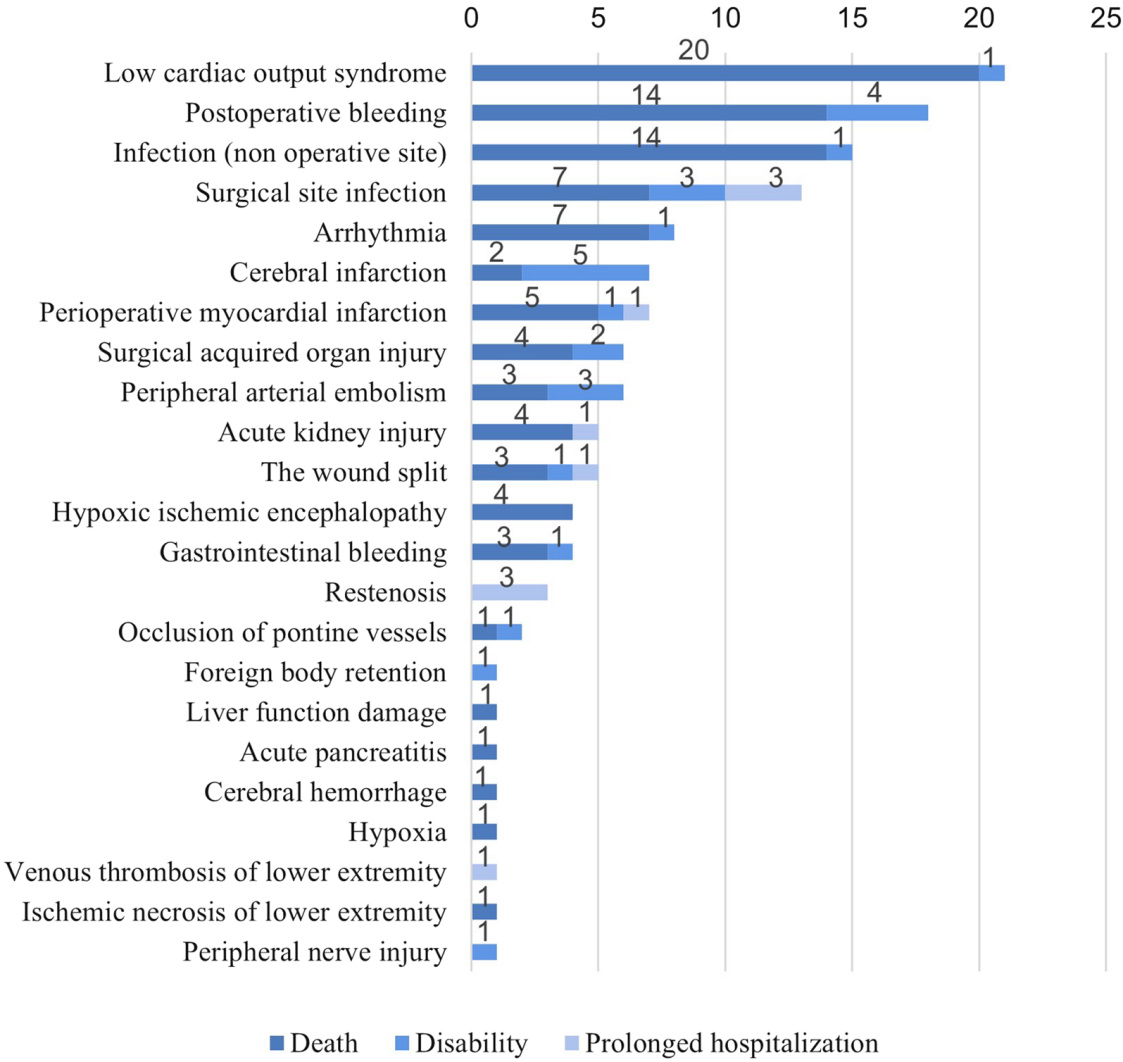
Cases distribution of postoperative complications in CABG medical claims according to patient outcomes. This figure reveals the number of cases with different patient outcomes (death, disability, extended hospitalization) for each postoperative complication in CABG medical claims

There were generally four types of patient outcomes involved in CABG medical claims cases: death, disability, prolonged hospitalization, and increased medical costs. In the sample data, only three cases were litigated because of the increase in medical expenses, which was due to transfusions resulting in blood-borne infectious diseases and harmless foreign body residues. Other litigation cases have basically involved the death, disability or prolonged hospitalization of patients due to postoperative complications. Therefore, postoperative complications can be considered as the direct cause of adverse outcome and patient prosecution. Quantitative analysis of the surgical complications involved in litigation cases can help clinicians assess the degree of liability they may bear when a patient develops such complications as a result of medical malpractice. The top 10 postoperative complications in dispute cases were: low cardiac output syndrome, postoperative hemorrhage, non-surgical site infection, surgical site infection, arrhythmia, cerebral infarction, perioperative myocardial infarction, organ injury, peripheral artery embolism, acute kidney injury. Cases distribution of postoperative complications in CABG medical malpractice litigations according to patient outcomes was shown in Fig. 2.

**Fig. 3:**
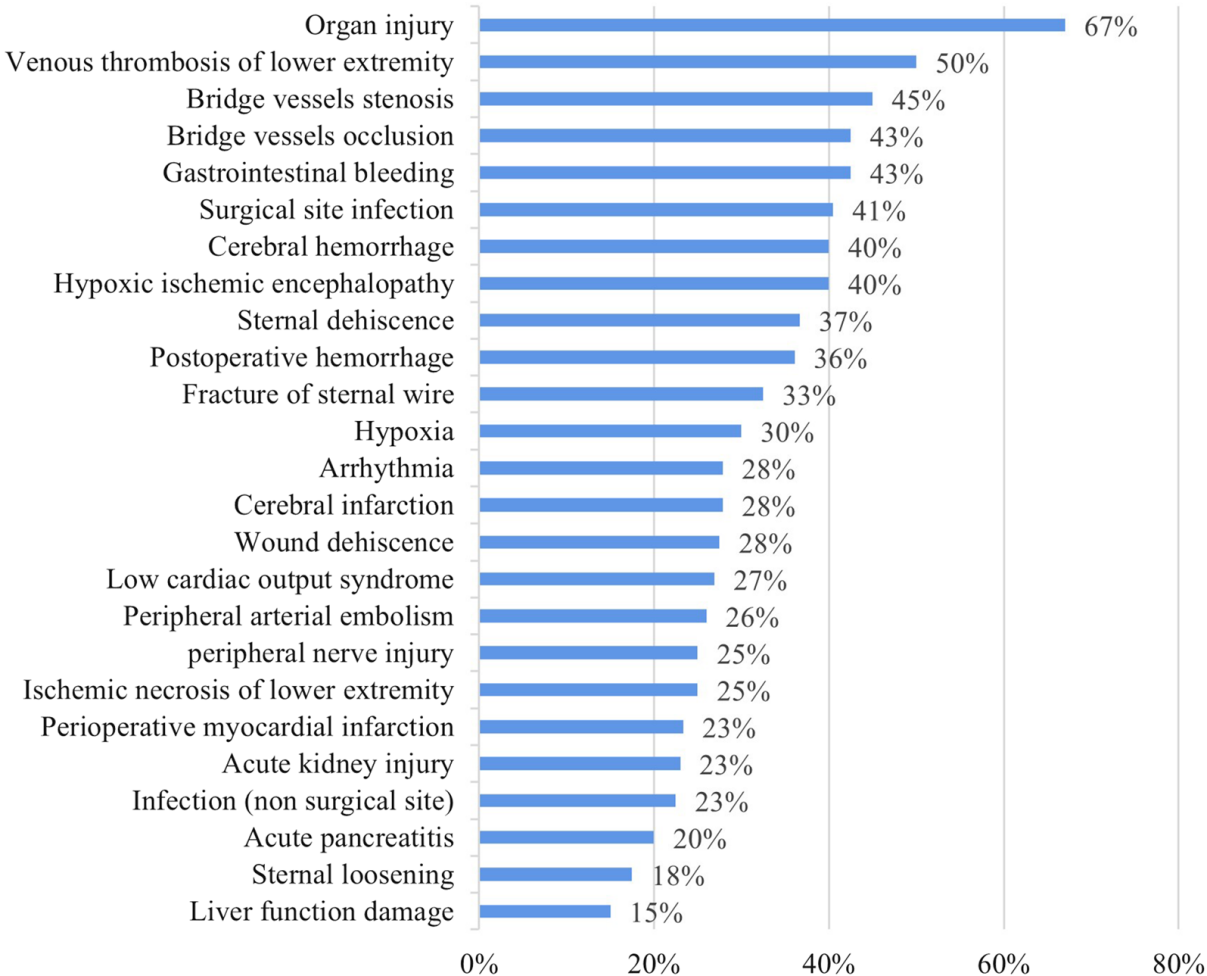
Proportion of liability of medical malpractice for postoperative complications. This figure reveals the Proportion of liability in each type of postoperative complications in CABG medical claims

The analysis of the proportion of liability of medical malpractice in the result of patient injury was meaningful to healthcare providers, which could provide clues to the improvement of medical care quality. In this study, the postoperative complication was used as a dimension to group the patient injury. The proportion of liability of medical malpractice for various postoperative complications was shown in Fig. 3. The common postoperative complication related to liability for compensation could be divided into the following categories: (1) Organ injury and postoperative bleeding caused by surgical procedures; (2) Wound management (wound and sternal fixation dehiscence, surgical site infection, etc.); (3) Neurological complications (cerebral hemorrhage, cerebral infarction, hypoxic ischemic encephalopathy); (4) Cardiac correlation (perioperative myocardial infarction, arrhythmia, low cardiac output syndrome, etc.); (5) Complications related to coagulation dysfunction in other systems (venous thrombosis of lower extremity, gastrointestinal bleeding, peripheral arterial embolism, etc.); (6) Acute kidney injury; (7) others. Complications related to insufficient basic operation (such as organ injury, bridge vessels stenosis, bridge vessels occlusion) and insufficient basic postoperative management (such as limb vein thrombosis, surgical site infection, cerebral hemorrhage, hypoxic-ischemic encephalopathy) seemed have a higher proportion of liability for compensation.

## Discussion

### Statement of principal findings

There were few recent researches on the characteristic of CABG malpractice claims in different level of hospitals. Our findings supported that the diagnosis and treatment capabilities of coronary artery bypass grafting in different levels of hospitals in China were inconsistent. The treatment capabilities of prefecture-level hospitals were lower than that of national hospitals because the proportion of responsibility for disputes related to municipal hospitals was higher than that of national hospitals, and the postoperative death time was shorter. Furthermore, the procedural error was the most common reason in CABG malpractice claims, which caused by surgical injuries have a higher proportion of liability.

### Strengths and weaknesses of the study

The current study systematically combed out the common medical quality issues in each medical procedure and quantitatively analyzed postoperative complications in CABG malpractice claims, while other studies usually conducted more generally categories in misadventure and rarely characterized postoperative complications leading to dispute cases, especially the proportion of related compensation liabilities.

Meanwhile, this study is subject to several limitations. First, cases of out-of-court settlement weren’t included resulting in a limited sample size, because the “China Judgments Online” database didn’t cover this type of cases. Second, the compensations had not been analyzed in this study. Since the claims occurred in different years and regions, the standardization of compensations was very difficult. Furthermore, the compensatory payment for a claim was determined by multiple factors, therefore calculating the damage for a single medical malpractice or a complication was difficult.

### Comparison with previous studies

The death of patients is one of the main causes of litigation. In this study the patient mortality occurred in 73.4% of litigations, while that was 49.2% (87 cases) in the medical malpractice litigations in Coronary Artery Bypass Grafting from 1994 to 2019 across the United States [12]. 53% of all cardiovascular claims and 61.81% of the paid claims were associated with the death of the patient, which were registered in the PIAA-DSP from 1985 to 2007 [13].

The most common basis for CABG litigation were procedural error, failure to properly monitor the patient and diagnostic errors, which was also observed in other surgical specialties [14–17]. No or delayed medical consultation, Improper operation scheme, improper surgical procedures, or surgical foreign body legacy was procedural error. Missed or false clinical examination, inadequate assessment, performing with contraindications or no indication or drug dose error was failure to properly monitor the patient. Diagnostic errors included failure to recognize or misdiagnose complications and comorbidities, which was a common reason for litigation [18]. The findings in our study were similar to that carried in the United States [19].

It was reported that surgery-related disputes were the most frequent in civil malpractice claims, and surgical complications was the top alleged reason [20]. Neurological injury and myocardial infarction were the most common clinical events, followed by infection, cardiac arrest, coronary artery dissection, prior insertion of a stent, emboli, congestive heart failure, stroke, and tapenade in the medical malpractice litigations in CABG from 1994 to 2019 across the United States. The low cardiac output syndrome, infection, cerebral infarction, myocardial infarction were reported as the common postoperative complications in this study as well.

Intraoperative negligence was significantly associated with poor defendant outcomes in rhytidectomy malpractice litigation [21]. The same was true in CABG malpractice claims. The proportion of liability for organ injury was the highest (64%), and that of foreign body retention, bridge vessels occlusion, postoperative hemorrhage and wound dehiscence all were above 30%.

### Meanings of the study

The insights gained from medical dispute cases may prompt surgeons pay more attention to high-risk links in the process of diagnosis and treatment, and facilitate identify near misses or significant events requiring root cause analysis. For example, there was a case of litigation due to bridge vessel stenosis after operation, which was judged by the court as violating the diagnosis and treatment routine, because the Transit-time flow measurement (TTFM) wasn’t performed during the operation. By learning this case, doctors would pay more attention to the importance of intraoperative graft assessment and standardize this procedure during surgery.

The medico-legal expert opinions recorded in the dispute judgment document analyzed the causal relationship between medical malpractice and the patient harm outcome in the medical dispute case. This study attempts to summarize the opinions of forensic doctors in similar cases and analyze the extent to which medical malpractice promote the development of patients' injuries. The analysis of the characteristics of medical quality problems in different levels of hospitals will help hospitals identify the direction of their quality improvement. The documents of medical malpractice verdicts, which was a kind of open access big data, would be further analyzed and applied with advances in natural language processing and artificial intelligence [22]. The judgment of the proportion of liability in medical disputes about surgical complications is a quantitative evaluation of their avoidability and severity in a sense, which can provide a reference for the hierarchical management of surgical complications.

## Conclusion

The diagnosis and treatment capabilities of coronary artery bypass grafting in different levels of hospitals in China were inconsistent, and the treatment capabilities in prefecture-level hospitals were lower than that in national hospitals. The procedural error, failure to properly monitor the patient and diagnostic errors were common in CABG claims. Postoperative complications related to insufficient basic operation and insufficient basic postoperative management lead to a higher responsibility proportion.

## Data Availability

All data generated or analyzed during this study are included in this published article.
